# Fatty acid composition in the vaginal tract of cis-gender women: canary in coal mines for reproductive health?

**DOI:** 10.1186/s12944-025-02489-7

**Published:** 2025-03-03

**Authors:** Karine Dufresne

**Affiliations:** Département des sciences biologiques, Université du Québec à Montréal, Montréal, Canada

**Keywords:** *Lactobacillus*, Vaginal tract, Fatty acids, Bacterial vaginosis, *S. Aureus*

## Abstract

The vaginal tract of cis-gender women of reproductive age is inhabited by communities of bacteria generally dominated by one of four *Lactobacillus* species. These bacteria are important for the reproductive health of women and favor better outcomes, including fertility, pregnancy leading to term and protection against infections. Past studies have focused on the role of carbohydrates in the balance of vaginal communities, and the role of fatty acids has been underestimated. However, small- to long-chain fatty acids present few properties that, in combination with sugar metabolism, affect the outcomes of the health or disease within the vaginal communities. Herein, we explore the origins of fatty acids in the vaginal tract as well as their roles in the bacterial life cycle in this environment. We also detail the putative impact of vaginal FAs on *S. aureus*, one of the etiologic agents of aerobic vaginitis. Finally, we discuss their potential for prevention or therapy in women of reproductive age.

The vaginal environment of cis-gender women of reproductive age is rich in nutrients that sustain microbial life, and these microorganisms are essential for women’s reproductive health. The vaginal microbiota is traditionally classified into community state types (CSTs), which are either dominated by specific L*actobacillus* species (CST-I = *L. crispatus*; CST-II = *L. gasseri*; CST-III = *L. iners*; CST-V = *L. jensenii*) or form a diverse community that is usually considered less optimal (CST-IV). CST-IV is associated with the presence of bacteria such as *Gardnerella vaginalis* (*G. vaginalis*), *Prevotella* species, *Porphyromonas* species and *Atopobium vaginae* (*A. vaginae*) [1]. Although CST-IV may evolve into bacterial vaginosis (BV), this CST is also present in several healthy women. However, CST-IV and CST-III are considered the less optimal vaginal communities. In the past few years, new efforts to understand and classify the vaginal microbiota have been made, and these studies expanded the diversity of the female community tested, leading to characterization of the vaginal microbiota worldwide [2, 3]. These studies demonstrated the importance of host factors in the composition of the microbiota, including age, pregnancy, sexual activity and individual habits (contraception, use of antibiotics, use of spermicides, and vaginal douching) [4–6]. Individual habits lead to changes in the vaginal environment, including changes in pH, oxygen levels, nutrients and metabolites [4]. Although traditional CSTs do not fully represent the diversity of the vaginal composition, they are simple models that recapitulate most of the bacterial signature.

It is unclear which microbe is the etiologic agent of BV. Few studies have noted the presence of *G. vaginalis* and A. *vaginae* in most cases of this disease, although we can associate the exfoliative properties of *Prevotella* and *Porphyromonas* species to BV as well [1, 7–10]. Another bacterial disease, aerobic vaginitis (AV), was described in 2002 by Donders et al. and has few known etiologic agents (*Staphylococcus aureus* [*S. aureus*], *Streptococcus agalactiae* [*S. agalactiae*], *Escherichia coli* [*E. coli*] and *Enterococcus faecalis* [*E. faecalis*]) [11, 12]. In rare situations, bacteria from the *Lactobacillus* genus may be in excess and accompanied by a large release of glycogen that leads to a disease named cytolytic vaginosis [13]. In addition to bacterial infections, other microorganisms lead to diseases, including the fungus *Candida albicans* (*C. albicans*), the etiologic agent of candidiasis, and several viruses (human immunodeficiency virus, human papillomavirus and hepatitis viruses) [14–17].

Vaginal secretions are mainly composed of mucin, fatty acids (FAs), salts, urea, iron chelators, and carbohydrates derived from glycogen [18–21]. Many vaginal changes rely on glycogen production by the vaginal epithelium and on the consumption of glucose, primarily by microorganisms. Epithelial cells produce glycogen, which is released by the cyclic exfoliation of this epithelium. Glycogen is then hydrolyzed by α-amylases and other enzymes (β-amylases, type-I and -II pullulanases) in glucose, which fulfills the most vital functions for the survival of microorganisms within the vaginal tract. All these processes occur in an environment with poor accessibility to oxygen, favoring fermentation over respiration for energy production [22]. Although we often associate colonization by beneficial *Lactobacillus* species in the vaginal tract with the presence of carbohydrates (glycogen and glucose), the importance of other molecules and their roles in colonization and signaling have yet to be studied under vaginal conditions. Moreover, these molecules may play a role not only in lactobacilli but also in pathogenic microorganisms of the female reproductive tract, leading to an intricate cascade of sensing and responding to their availability. Herein, we explore the origin and role of FAs in bacterial metabolism, survival, signaling and virulence within the vaginal niche, especially their importance in maintaining or disrupting women’s reproductive health.

## Origins of lipids and fatty acids in the reproductive system of women

FAs have two main origins: they are either host-derived (mainly long-chain fatty acids or LCFAs) or produced through bacterial metabolism. The human mucosa (i.e., skin, nares, intestinal tract, and vaginal tract) tends to be rich in LCFAs, which are specifically secreted by the host for their antibacterial properties or found through cellular degradation [23–29]. Polyunsaturated LCFAs with *cis* double bonds are especially potent antimicrobial agents [30]. The concentration and composition of LCFAs in vaginal fluids is still poorly known. Nevertheless, the lipidic pool in the vagina during pregnancy is markedly associated with glycerophospholipid, sphingolipid and ether lipid metabolism suggesting a possible degradation of these lipids and the presence of C16 and C18 FAs such as palmitic acid or oleic acid [29].

The majority of short-chain fatty acids (SCFAs) within the vaginal tract stem from microbial metabolic activities, especially carbohydrate fermentation and amino acid catabolism [31]. The vaginal environment is rich in glycogen, a large polymer of glucose connected through α-1,4- and α-1,6-glycosidic bonds. Glycogen is hydrolyzed into smaller sugars by human and microbial enzymes [32, 33]. Among these enzymes, α-amylases facilitate the optimal degradation of glycogen via the hydrolysis of α-1,4-glycosidic linkages, which results in the production of maltose, maltotriose and α-limit dextrins [34]. Although women produce their own α-amylases, these enzymes lose the majority of their activity at acidic pH [32]. Thus, human α-amylases are hypothesized to be the initial enzymes that catabolize the glycogen accumulation starting at puberty and then allows the establishment of *Lactobacillus* species within the vaginal tract [32]. Compared with amylases produced by microorganisms residing in the vaginal tract, the involvement of human amylases from menarche is weak [32]. Interestingly, few vaginal bacterial species produce enzymes such as 1,6-glucosidases and pullulanases [32, 35]. These enzymes degrade glycogen mainly into maltotetraose [35]. Among the resident bacteria, *L. crispatus* and *L. iners* produce similar enzymes and cleave both α-1,4- and α-1,6-glycosidic bonds, although 20% of the *L. crispatus* population encodes a dysfunctional PulA [32, 36]. *L. gasseri* and *L. jensenii* produce mainly glucosidases that cleave α-1,6-glycosidic bonds [32]. In addition to *Lactobacillus*, *G. vaginalis* also produces a pullulanase [37]. The processes of carbohydrate degradation are of special importance for the subsequent metabolic steps occurring within the vaginal tract, leading either to the production of lactic acid by the *Lactobacillus* species or to the production of SCFAs by the less stable communities.

Lactic acid (lactate) is the main metabolite found in the vaginal tract of reproductive-age women [38]. The vaginal tract is an environment limited in oxygen, which promotes the use of alternative respiratory pathways by *Lactobacillus* species, including homolactic and heterolactic fermentation [39, 40]. Fermentation is responsible for the production of most lactic acid and acidification of the vaginal tract to ~ pH 4, except during menstruation when the pH reaches near neutral [41, 42]. Most of the beneficial effects are associated with D-lactic acid, and *Lactobacillus* species that produce better concentrations of D- versus L-lactate present better stability and better health outcomes [13, 43, 44]. Although most lactate production is associated with bacteria, the vaginal mucosa also uses anaerobic fermentation, leading to the production of a small proportion of lactic acid within the vaginal tract [45–47].

Conversely, in communities that are not dominated by *Lactobacillus* species, the FA composition changes drastically. When the lactate concentration decreases, SCFA levels increase, favoring the development of vaginal dysbiosis [15]. This shift from lactic acid to increased concentrations of other SCFAs, including acetate, propionate, succinate and butyrate, is one of the hallmarks of bacterial vaginosis (BV) [15]. Acetate and succinate are increased in symptomatic BV patients, however this production of SCFAs was not associated with any specific BV bacteria so far [15, 48]. *L. jensenii* is also able to produce a significant amount of acetate in addition to its production of lactic acid; however, the balance between lactic acid and acetate is still maintained for this *Lactobacillus* specie [49, 50]. Interestingly, the relationship between SCFA production and BV is not clearly understood.

**Fig. 1:**
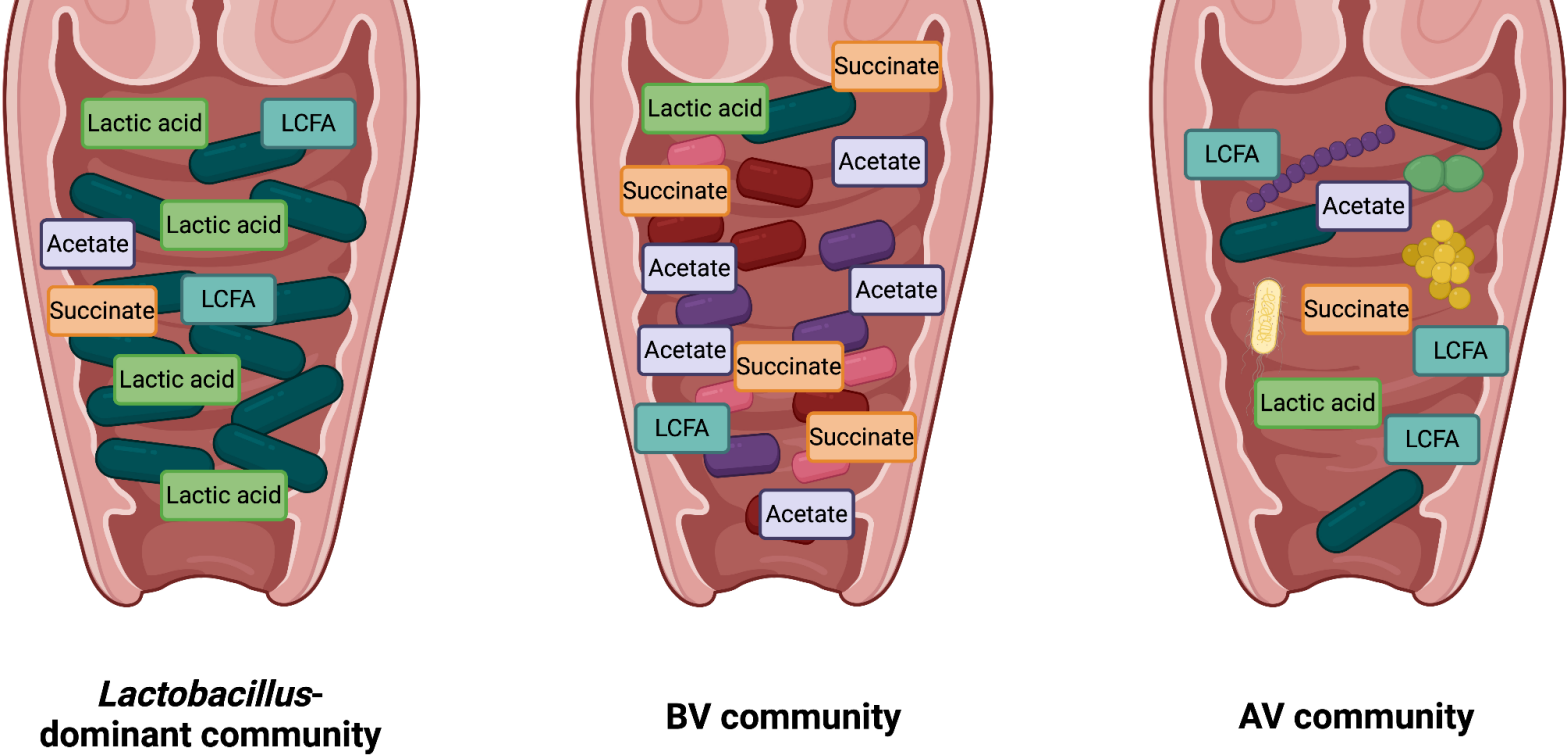
FA composition and vaginal communities. The main FAs found in *the Lactobacillus*-dominant, BV or AV community are represented in this figure. In an optimal *Lactobacillus*-dominant community (CST-I, -II or -V), it is expected that the concentration of lactic acid will be greater than SCFAs (acetate and succinate). LCFAs are present and provide an additional mechanism for *Lactobacillus* dominance. In a BV-prone community, fewer *Lactobacillus* species are present, leading to a decrease in lactic acid, and BV bacteria produce SCFAs, especially succinate and acetate. LCFAs would also be present. In the AV-prone community, little is known. This community is believed to be more similar to the BV-prone community

A similar decrease in lactate concentration is found in other bacterial infections, including infection by *Chlamydia trachomatis* (*C. trachomatis*), the agent of chlamydia, a sexually transmitted disease [51]. In this case, the lactate decrease is mainly associated with a switch from vaginal *Lactobacillus* species to a population dominated by *L. iners* instead [51]. Given that AV is still a recent diagnosis, no study has focused on the FA profile in this context; it is likely that changes similar to BV will be observed as the AV bacteria disturb the vaginal communities and lead to a disruption of the *Lactobacillus* dominance as well [12, 52]. Overall, an interesting component of most bacterial infections in the female reproductive tract is this modification of the lipidic landscape of the vaginal mucosa. These microorganisms also interfere with lactate production by lactobacilli [53]. To date, little is known about the lipid signature of vaginal infections, and this gap needs to be filled to find new therapeutic strategies. Figure 1 synthesizes the known and hypothesized composition in FAs within the vaginal tract.

## Microbial survival and response to sensing fatty acids within the vagina

FA-producing microorganisms use these metabolites for interbacterial and interkingdom competition with certain species in the vaginal environment [14, 27, 53]. During microbial interactions, FAs fulfill several roles, including antimicrobial (LCFA and medium-chain FA) or antivirulent functions as well as signaling for microbial response [54]. Additionally, in the case of carbohydrate starvation, FAs become a source of carbon and change the metabolic pathway (e.g., β-oxidation) of bacteria that are able to survive within these restrictions [55]. Herein, we focus on the role of FAs as antimicrobial molecules and as signals for the regulation of vaginal bacteria.

Among the FAs, oleic acid, linoleic acid and palmitoleic acid have shown protective effects on *Lactobacillus*-dominant communities, which are considered relatively stable; however, these LCFAs are detrimental to *L. iners* [27]. Two major determinants explain the observed differences: an oleate dehydratase (OhyAB) and an efflux pump (FarE) in strains of *L. crispatus*, *L. gasseri* and *L. jensenii* but not in *L. iners* [27, 56]. In addition to the resistance of vaginal *Lactobacillus* species to oleic acids and derivates, these unsaturated LFCAs promote their dominance within the vaginal tract, suggesting that these host-derived FAs are important for the maintenance of *Lactobacillus* and therefore protection of the niche [27]. Indeed, the vaginal environment is specifically designed to provide essential nutrients to the vaginal *Lactobacillus* species and to fight alongside these bacteria against less favorable microbes through the production of antimicrobials such as FAs.

Conversely, oleic acid and its derivatives inhibit bacteria with suboptimal health outcomes in the vagina (i.e., BV bacteria and *L. iners*) [27]. BV-associated bacteria also lack OhyA and FarE, similar to *L. iners*, and do not present any predicted oleate dehydratase-encoding genes [27]. These bacteria are unable to detoxify or integrate host-derived LCFAs into their membranes, and the presence of these metabolites instead provokes lysis of bacterial cells [27]. Several possibilities for survival in the presence of host LCFAs must be envisioned, including the activation of the immune response in the presence of BV-prone bacteria and the production of BV-prone virulence factors in response to stress. The bacteria associated with BV are known to trigger a host response via many mechanisms, including the production of virulence factors and specific SCFAs [1, 57–60].

**Fig. 2:**
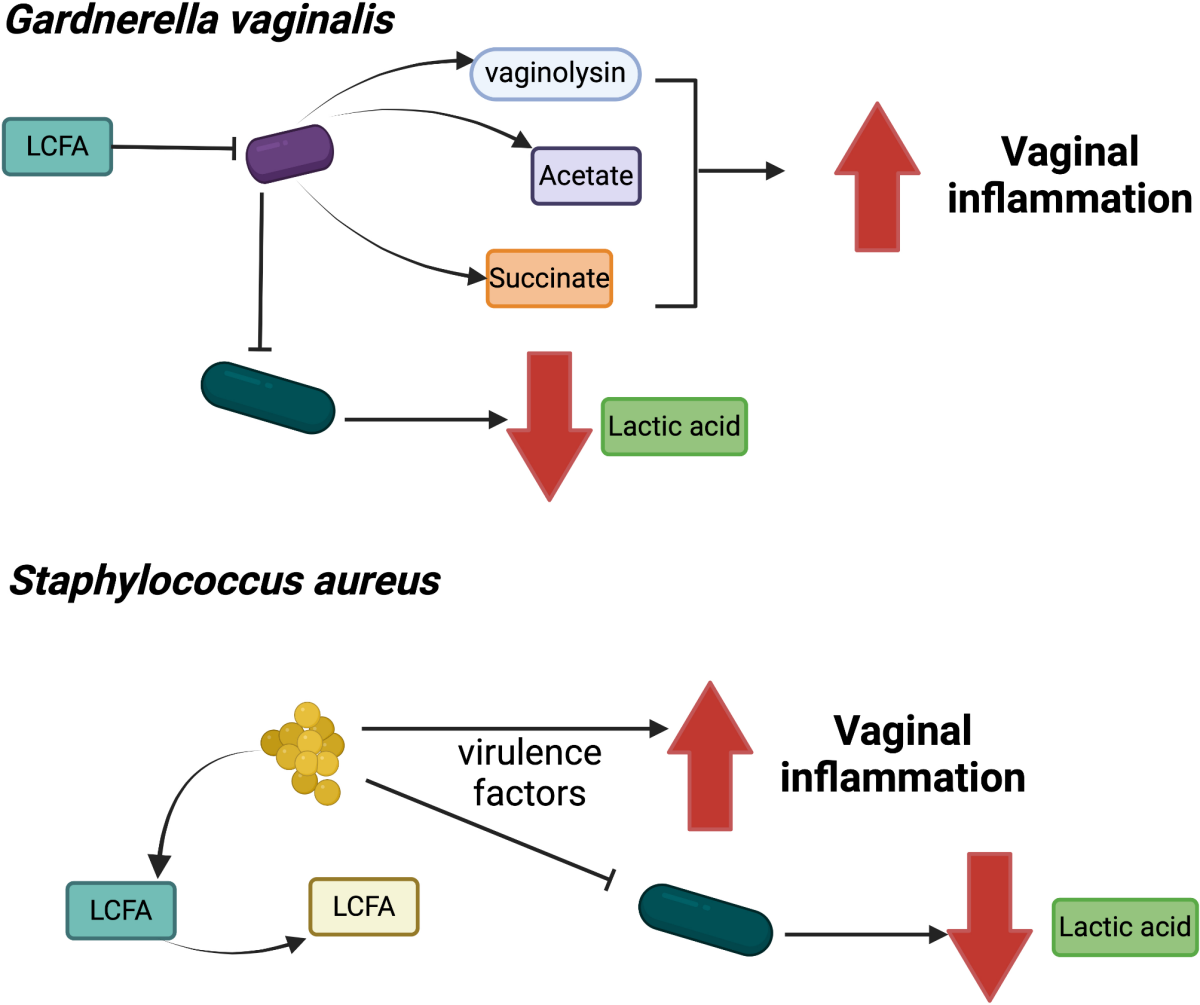
FAs in interaction with BV or AV bacteria. A synthesis of the main findings on mechanisms of interaction between FAs and prototypic bacteria is presented. *G. vaginalis* (purple bacterium), a BV-associated bacterium, is unable to modify and integrate LCFAs like oleic acid and their growth is normally inhibited in their presence. However, through sensing stressful signals and responding by activation of virulence factors (e.g. vaginolysin) and by producing acetate and succinate, *G. vaginalis* is triggering vaginal inflammation. The mechanisms of action are still unclear for these changes as well as for the mechanisms that lead to the switch from *Lactobacillus* (dark blue bacteria) dominance to a diverse community (BV-prone). *S. aureus* (golden spherical bacteria), an AV-prone bacterium, can modify certain LCFAs by the action of OhyA and FakA, alleviating the toxicity of these host-derived FAs and allowing its survival. In response to stress, *S. aureus* will produce virulence factors including TSST-1. These virulence factors are responsible for triggering vaginal inflammation and for the modification of the vaginal community leading to a decrease of lactic acid production

Although no unique etiologic agent has been defined for BV, *G. vaginalis* and *Prevotella* species (especially *P. bivia*) are generally the two most prominent bacteria prone to this disease [1]. Among the known virulence factors of *G. vaginalis*, the bacterium has the capacity to produce biofilm, adhere to the epithelium and produce cytotoxic components [61, 62]. Interestingly, this bacterium produces vaginolysin, which activates the production of interleukin-8 from human epithelial cells and is considered the main determinant of its pathogenicity (Fig. 2) [63]. In the case of *Prevotella* and *Porphyromonas* species, their proteolytic activities have been recently shown to disrupt vaginal communities through activation of the proinflammatory response [7, 8]. Little is known about how virulence factors are regulated in *G. vaginalis*, *Prevotella* and *Porphyromonas*, and even less is known about their role in the vaginal environment. Therefore, we hypothesize that extracellular proteins (vaginolysin and proteases) are activated in the vaginal tract in response to stress signals within this environment. In addition to the virulence factors impacting the vaginal mucosa, the BV-prone community presents a decreased concentration of lactic acid through disruption of the *Lactobacillus* dominance leading to the inhibition of anti-inflammatory signals associated with D-lactate [40, 48]. Additionally, the BV bacteria produce high levels of acetate and succinate that are suggested to increase TLR4 expression leading to production of pro-inflammatory cytokines [48, 53, 64]. Indeed, the regulation of metabolism and virulence within BV-prone bacteria is still poorly understood [65]. Hence, future studies to understand the dynamism of the vaginal tract should focus on how these bacterial species sense and respond to vaginal cues.

Like BV, several bacteria are associated with AV, and no unique etiologic agent has been defined. However, most AV-prone bacteria are opportunistic pathogens and are also found on other mucosal surfaces [12, 66]. Both *E. coli* and *S. aureus* have been extensively studied for their survival in response to host-derived FAs in other organs. Both bacteria have several mechanisms for detoxifying and metabolizing FAs; these mechanisms were recently reviewed by Kengmo Tchoupa et al., 2022 [56].

*S. aureus* is especially interesting for the interaction with FAs in the vaginal tract. Several studies have demonstrated few mechanisms for *S. aureus* to survive in the presence of host-derived FAs on the skin, the main colonization site of the bacterium [55, 56, 67–72]. Herein, we apply to the vaginal tract, the knowledge researchers have built about interactions between *S. aureus* and FAs in other niches. *S. aureus* uses fatty acid kinase A (FakA) to incorporate exogenous FAs into its membrane and assure the integrity of the bacterial cell [69, 70, 73–75]. This mechanism would also compensate for the loss of its FA synthesis system (FASII) in the case of inhibition of this pathway [76]. Otherwise, the bacterium uses the fatty acid-modifying enzyme (FAME), recently identified as the lipase Lip2, to detoxify the host-derived FAs in addition to OhyA and efflux pumps [67, 77–79]. Taken together, bacteria use several detoxification and metabolic pathways to survive and thrive in the presence of host-derived FAs at physiological concentrations, and these mechanisms are regulated mainly by GraXRS (Fig. 2) [56, 80].

Like other vaginal bacteria, FAs are responsible for the regulation of *S. aureus*; however, many regulators impact virulence factors in the vaginal tract. One major virulence factor that has been studied in this environment is toxic shock syndrome toxin-1 (TSST-1), which is responsible for menstrual toxic shock syndrome (mTSS), a rare but life-threatening disease affecting young women of reproductive age [66, 81, 82]. As its name suggests, mTSS occurs at menstruations and is associated with the use of menstruation management devices such as tampons or menstrual cups [82–85]. The production of the toxin is tightly regulated it is mainly repressed by the presence of glucose in the vaginal tract [86]. Otherwise, the introduction of oxygen at the insertion of the earlier generation of tampons, in combination with changes in other environmental cues, triggers the production of an important concentration of TSST-1, leading to the clinical outcomes of mTSS [86–90]. Although mTSS represents the most acute condition in the vaginal tract, studies on the regulation of TSST-1 highlight the importance of maintaining a balance between *S. aureus*, the host and the microbiota, especially in an environment as dynamic as the vaginal tract. In this context, we believe that several other regulators are involved in the production of toxins and other colonizing factors important for *S. aureus*, including iron-regulated surface determinant proteins (Isd) and fibrinogen-binding proteins [91, 92]. Among the putative regulatory and survival mechanisms, the two-component system GraXRS possibly senses the acidic environment within the vagina and might also regulate *S. aureus* virulence factors and resistance to host-derived FAs in this context [80]. This system remains to be studied in the vaginal context to understand its role for the intricate regulation of *S. aureus* in this specific environment. *S. aureus* is a great example of a colonizing bacteria that is able to trigger pathogenicity in response to changes in its environment, and more efforts are needed to obtain a full picture of the mechanisms leading to colonization or diseases such as AV or mTSS [66].

## Fatty acids as determinants of a healthy microbiota

In the vaginal tract, the composition of FAs is a consequence of changes in the community and in metabolic activities. BV and AV diagnoses are still made on the basis of microscopic examination; thus, FAs (mainly acetate and succinate) could be used as biomarkers to facilitate the validation of BV. Although this review did not explore the role of FAs in candidiasis, there are significant differences in the lipidic signature during this disease as well as their impact on *C. albicans* pathogenesis (growth, biofilm formation and morphogenesis), adding to our conclusion that the lipidic signature of the vagina is a determinant clue leading to the diagnosis of microbial infections beyond BV [14, 93–96]. Moreover, the findings from the vaginal environment are of great interest as FAs, more precisely SCFAs, play a converse role for the intestinal microbiota where their presence is generally associated with healthy outcomes [97]. Interestingly, D-lactic acid also demonstrate converse role in the gut microbiome suggesting a niche-specific distribution of roles associated either with the microbial population or with the specificity of the host tissue [98]. In the future, investigating the specific changes in FAs occurring in microbial infections and comparing these FA patterns between diseases, organs and even between etiologic agents for a more accurate diagnosis will be of outmost importance.

In addition to their potential in the prediction of diseases within the reproductive tract of cis-gender women, FAs, especially LCFAs, show great potential for therapy for combination with antibiotics [54]. Most host-derived FAs are already known for their antimicrobial activities [54, 79]. The example of oleic acid and its derivatives in in vitro experiments with representative strains from the vaginal community showed a real advantage given to *Lactobacillus* species associated with health compared to *L. iners* and *G. vaginalis* [27]. However, certain opportunistic pathogens associated with AV easily manipulate FAs, evade these antimicrobial metabolites and become more resistant [67, 70, 76]. A better understanding of the metabolic pathways of FAs and their impact on gene regulation in vaginal bacteria will allow the use of these metabolites for future treatment.

## Data Availability

No datasets were generated or analysed during the current study.

## Abbreviations

AV: Aerobic vaginitis
BV: Bacterial vaginosis
*C. albicans*: *Candida albicans*
*C. trachomatis*: *Chlamydia trachomatis*
CST: Community state type
*E. coli*: *Escherichia coli*
*E. faecalis*: *Enterococcus faecalis*
FA: Fatty acid
FakA: Fatty acid kinase A
FAME: Fatty acid-modifying enzyme
FASII: Fatty acid synthesis system
*G. vaginalis*: *Gardnerella vaginalis*
Isd: Iron-regulated surface determinant proteins
*L. crispatus*: *Lactobacillus crispatus*
*L. gasseri*: *Lactobacillus gasseri*
*L. iners*: *Lactobacillus iners*
*L. jensenii*: *Lactobacillus jensenii*
LCFA: Long-chain fatty acid
mTSS: Menstrual toxic shock syndrome
*P. bivia*: *Prevotella bivia*
*S. aureus*: *Staphylococcus aureus*
*S. agalactiae*: *Streptococcus agalactiae*
SCFA: Short-chain fatty acid
TSST-1: Toxic shock syndrome toxin-1
